# Tissue properties and respiratory kinematics of the tongue base and soft palate in the obese OSA minipig

**DOI:** 10.1371/journal.pone.0293907

**Published:** 2023-12-07

**Authors:** Daniel F. Leotta, Daniel Ly, Bishoy Galil, Jeff Thiel, Elliot Willis, Niranjan Balu, Zi-Jun Liu

**Affiliations:** 1 Center for Industrial and Medical Ultrasound/Applied Physics Laboratory, University of Washington, Seattle, WA, United States of America; 2 Dept. Orthodontics, School of Dentistry, University of Washington, Seattle, WA, United States of America; 3 Dept. Radiology, School of Medicine, University of Washington, Seattle, WA, United States of America; University of Enna Kore: Universita degli Studi di Enna ’Kore’, ITALY

## Abstract

Obesity is a common finding and a major pathogenetic factor in obstructive sleep apnea (OSA) in adults. To understand the mechanisms behind this, the present study investigated the tissue properties and respiratory kinematics of the tongue base and soft palate in the obese OSA minipig model. In 4 verified obese/OSA and 3 non-obese/non-OSA control minipigs, MRI fat-weighted images, ultrasound elastography (USE), and sleep video-fluoroscopy (SVF) were performed to quantify the fat composition, tissue stiffness, and respiratory kinematics of the tongue base and soft palate during sedated sleep. The results indicated that the fat composition gradually increased from the rostral to caudal tongue base, particularly in the posterior 1/3 of the tongue base, regardless of the presence of obesity and OSA. However, this trend was not seen in the soft palate and pharyngeal wall. The pharyngeal wall presented the highest fat composition as compared with the tongue base and soft palate. Overall, obese OSA minipigs showed stiffer tongue tissue than the controls, particularly in the rostral region of the tongue in obese Yucatan minipigs. The respiratory moving ranges of the soft palate were greater in both dorsal-ventral and rostral-caudal directions and during both respiratory and expiratory phases in OSA obese than control minipigs, and the largest moving ranges were seen in OSA obese Panepinto minipigs. The moving range of the tongue base was significantly smaller. These results suggest more fat infiltration in the caudal region of the tongue base regardless of the presence of obesity and/or OSA. The greater tissue stiffness of the tongue in obese OSA minipigs may result from altered neuromuscular drive.

## Introduction

Obstructive sleep apnea (OSA) is a respiratory disorder characterized by the repetitive collapse of the oropharyngeal airway during sleep. The muscle tone of the tongue, soft palate, and pharyngeal wall becomes relaxed in sleep, which may cause partial or complete collapse of the oropharyngeal airway, thus impeding airflow. Lingual and pharyngeal tonsils and/or adenoid hypertrophies may also cause these problems, especially in children [1]. OSA affects up to 20% of the population and leads to repetitive disruption of sleep and sleep deprivation. OSA is therefore a major risk factor for fatigue related accidents and cardiovascular diseases, and severe OSA can even lead to death [2]. Craniofacial deformity, brain injury, decreased muscle tone and aging have all been linked to OSA, but the biggest risk factor is thought to be obesity [3]. A study conducted by de Sousa *et al*. suggested that an increase of 6 units of body mass index (BMI) led to a fourfold risk of developing OSA [4]. On the other hand, the morphology and tissue composition of the oropharyngeal structures, such as the tongue, the soft palate, and the pharyngeal wall, may affect their biomechanical properties, which could contribute to the pathology of OSA as well. A study conducted by Bonsignore *et al*. suggested that there are fundamental differences in the biomechanical tissue properties of the tongue in patients with OSA compared to that of patients without OSA, and these changes are not the result of only age or obesity but also genetics [5]. Of course, the variation of anatomical districts may alter these biomechanical tissue properties [6, 7]. An analysis of sleep status further suggested that obesity (BMI > 30) increases the severity of OSA in military personnel diagnosed with OSA [8]. However, the inherent association between OSA and obesity is not well understood.

By using obese/OSA and normal control minipig models, the goal of the present *in vivo* study is to quantify the adipose tissue proportions in the tongue base, soft palate, and oropharyngeal wall, to examine the biomechanical properties of different regions of the tongue and neck muscles, and to digitize the trajectory of the respiratory excursions in the tongue base and soft palate. The working hypothesis is that compared with normal control minipigs, obese/OSA minipigs would have the following three features: 1) increased accumulation of adipose tissue in the tongue base, soft palate, and oropharyngeal wall; 2) altered tissue stiffness of the tongue due to more infiltration of adipose tissue and/or modified neuromuscular physiological status; and 3) extended trajectory of the respiratory movements in the tongue base and soft palate to enhance the airflow passing the obstructed airway.

## Materials and methods

### OSA verifications

The present study included 7 minipigs: 5 Yucatan minipigs aged 8–11 months (Premier BioSource, Ramona, CA) and 2 Panepinto minipigs aged 6.5 years (Panepinto & Associates, CL). All procedures were adhered to the Animal Research Reporting of *In Vivo* Experiments (ARRIVE) guidelines and approved by Institutional Animal Care and Use Committee (IACUC) of University of Washington (Protocol# 3393–04).

Of the 5 Yucatan minipigs, 3 were non-obese controls with BMI ranging from 37.93 to 38.91, and 2 were obese with BMI of 50.13 and 51.42. The two Panepinto minipigs were both obese with BMI of 48.75 and 59.30 (**Table 1**). The rationale for the obesity determination and development was published elsewhere [9]. After 5–7 day reverse lighting cycle training and acclimation to wear the jacket that was used to hold the sleep recording devices, all 7 pigs received live sleep monitoring for 3–4 hours using two sets of wireless BioRadio systems (Great Lakes NeuroTechnologies, OH) to record the following physiological parameters during natural sleep: 1) respiratory airflow and snoring; 2) chest and abdominal respiratory movements; 3) oxygen saturation; 4) electroencephalography (EEG) at the sites of C3 and C4; 5) bilateral electrooculography (EOG); and 6) electromyography (EMG) of the right middle pharyngeal constrictor muscle. The analyses of these sleep recordings indicated that the apnea or hypopnea indexes (AHI) were 0.00–4.60 in 3 controls. Heavy snoring during sleep occurred in all 4 obese minipigs and AHIs were 30.00 and 32.86 for the two obese Yucatan, and 58.70 and 67.60 for the two obese Panepinto minipigs (**Table 2**). The detailed definition of AHI and verification of OSA episodes were published elsewhere [9].

**Table 1:** Physical characteristics of minipigs.

Pig No.	Age	Sex	BW(Kg)	BL(cm)	BMI(Kg/m²)	NC(cm)
**Non-obese Yucatan**						
716	8.0 M	F	45.90	110.00	37.93	61.00
970	9.5 M	M	55.00	120.00	38.19	66.00
981	11.0 M	F	55.10	119.00	38.91	74.00
**Obese OSA Yucatan**						
930	8.5 M	M	68.00	115.00	51.42	82.00
954	8.5 M	F	71.00	119.00	50.13	77.00
**Obese OSA Panepinto**						
7	6.5 Y	M	85.60	132.00	48.75	82.00
10	6.5 Y	M	104.10	132.00	59.30	94.00

**BW:** body weight.

**BL:** body length; measured from the tip of snout to the base of tail.

**BMI**: body mass index, calculated as body weight (Kg) / body length (m)^2^.

**NC:** neck circumference, measured at the location of thyroid cartilage.

**Table 2:** Comparisons between natural and sedated sleep.

	RR (minute)		TV (ml)		HR (minute)	
Pig No.	N	S	N	S	N	S
**Non-obese Yucatan**						
716	n/a	12–28	n/a	300/300	n/a	63–86
970	n/a	27–41	n/a	200/300	n/a	69–91
981	n/a	14–23	n/a	400/400	n/a	45–57
**Obese OSAYucatan**						
930	20–24	23–31	700/300	300/300	75–81	80–85
954	20–25	25–35	900/650	700/400	79–81	78–82
**Obese OSAPanepinto**						
7	n/a	12–19	n/a	1300/700	n/a	n/a
10	15–21	19–26	1,900/1,500	1,800/1,300	n/a	77–84

**RR:** Respiratory rate; **TV:** Tidal volume, inspired TV/expired TV; **HR:** Heart rate

**N & S:** Natural & Sedated sleep.

**n/a:** not available

### Image acquisitions

Images of MRI, ultrasound elastography (USE), and sleep video-fluoroscopy (SVF) were all obtained during sedated sleep in three separate sessions.

#### MRI–Fat composition

**Fig 1:**
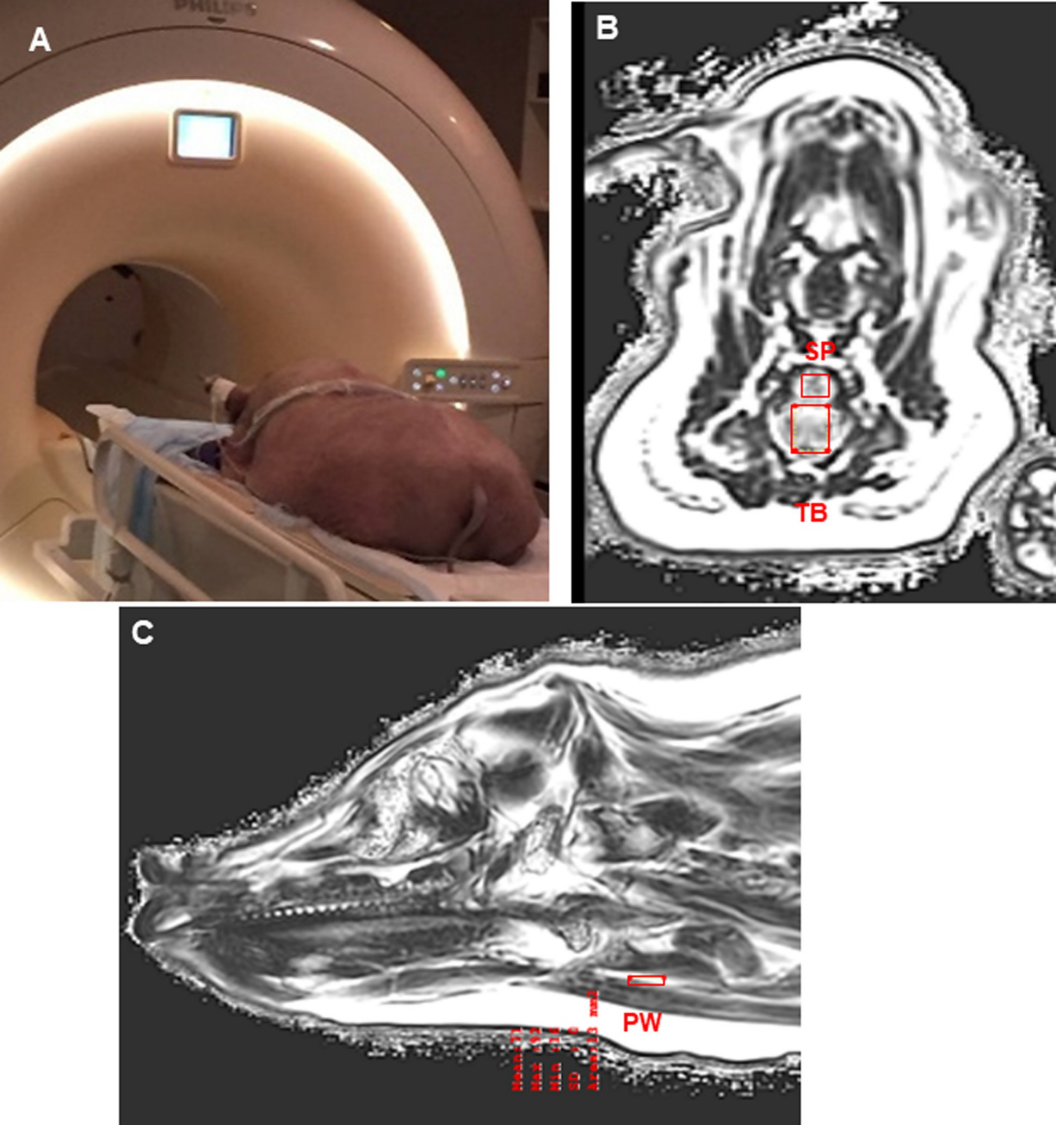
MRI imaging and fat composition measurements. **A:** Live MRI scanning. **B:** Coronal view of MRI Dixon fat water separated sequences. The two red boxes are the regions for the fat composition measurement of the tongue base (TB) and soft palate (SP). The measurements were taken in the serial 8 coronal slices from the most rostral to the most caudal with thickness of 5mm in the tongue base and soft palate. **C:** Sagittal view of MRI Dixon fat water separated sequences. The red box shows the region for the fat composition measurement of the pharyngeal wall (PW). The measurements were taken in the serial 3 sagittal slices from the mid-sagittal to the lateral and internal with thickness of 5mm.

MRI images (3T Philips Ingenia Quasar Dual 3.0T whole body scanner) with T1 weighted Volumetric Isotropic Turbo spin echo Acquisition (VISTA) and Dixon fat water separated sequences were taken with the minipig placed in the prone position within the scanner bore (the most common sleep position in pigs (**Fig 1A,** MRI imaging and Fat Composition measurements. **A:** Live MRI scanning. **B:** Coronal view of MRI Dixon fat water separated sequences. The two red boxes are the regions for the fat composition measurement of the tongue base (TB) and soft palate (SP). The measurements were taken in the serial 8 coronal slices from the most rostral to the most caudal with thickness of 5mm in the tongue base and soft palate. **C:** Sagittal view of MRI Dixon fat water separated sequences. The red box shows the region for the fat composition measurement of the pharyngeal wall (PW). The measurements were taken in the serial 3 sagittal slices from the mid-sagittal to the lateral and internal with thickness of 5mm).

#### USE–Tissue stiffness

**Fig 2:**
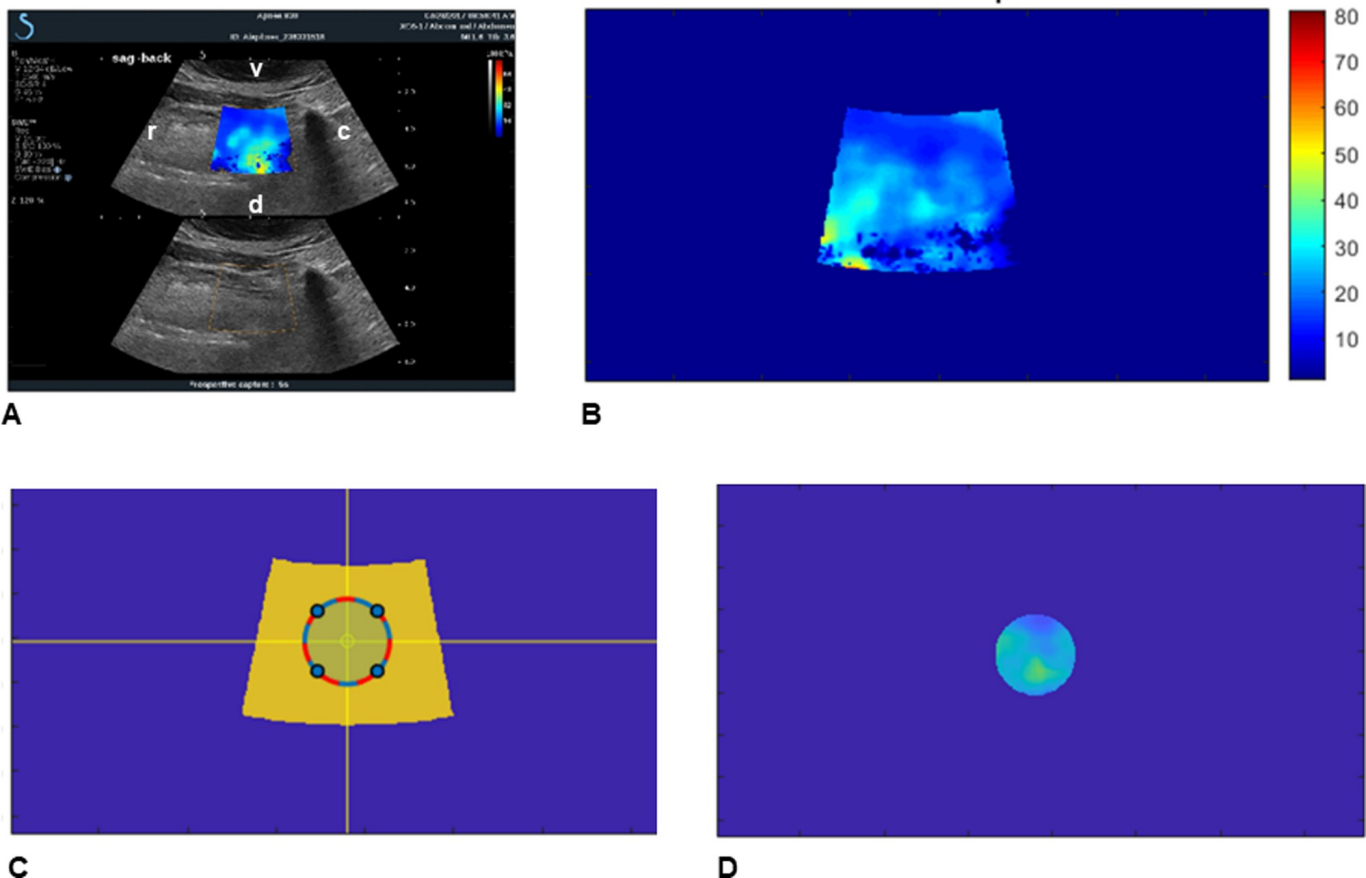
Ultrasound elastography (USE). **A:** Sagittal USE image of the tongue. The elastography measurement region is shown with color overlay in the top image. The dark region on the right side of the image is due to shadowing from the hyoid bone. **B:** Colorized ROI with scale bar converting color to stiffness value (kPa); **C:** Circular ROI from the center of the USE color region. **D:** The stiffness measurement according to the color scale in **B** for the extracted circular ROI. **r:** rostral, **c:** caudal; **v:** ventral; **d:** dorsal.

USE data [10, 11] were collected with an Aixplorer scanner from Supersonic Imagine (Aix-en-Provence, France). USE imaging was performed with a curvilinear scanhead with a frequency bandwidth of 1–6 MHz (SSI transducer XC6-1). The ultrasound probe was placed in the submandibular region with sagittal orientation in the regions of rostral, middle and caudal tongue (**Fig 2A,** Ultrasound Elastography (USE). **A:** Sagittal USE image of the tongue. The elastography measurement region is shown with color overlay in the top image. The dark region on the right side of the image is due to shadowing from the hyoid bone. **B:** Colorized ROI with scale bar converting color to stiffness value (kPa); **C:** Circular ROI from the center of the USE color region. **D:** The stiffness measurement according to the color scale in **B** for the extracted circular ROI. **r:** rostral, **c:** caudal; **v:** ventral; **d:** dorsal**)** and at the left neck muscles. This ultrasound instrument uses a specialized scanning mode for non-invasive measurement of tissue stiffness known as Shear Wave Elastography (SWE). SWE uses a high-powered acoustic pulse to create a shear wave in the tissue of interest, which is a pressure wave that propagates laterally across the image field of view (perpendicular to the propagation direction of the standard imaging pulses). Imaging with extremely high frame rates (typically several thousand frames per second) allows the instrument to track the lateral propagation speed of the shear waves as they pass through tissues. These propagation speeds, which vary as the shear waves pass through different tissue types, are used to calculate regional tissue stiffness (**Fig 2A**).

#### SVF–Moving trajectories

**Fig 3:**
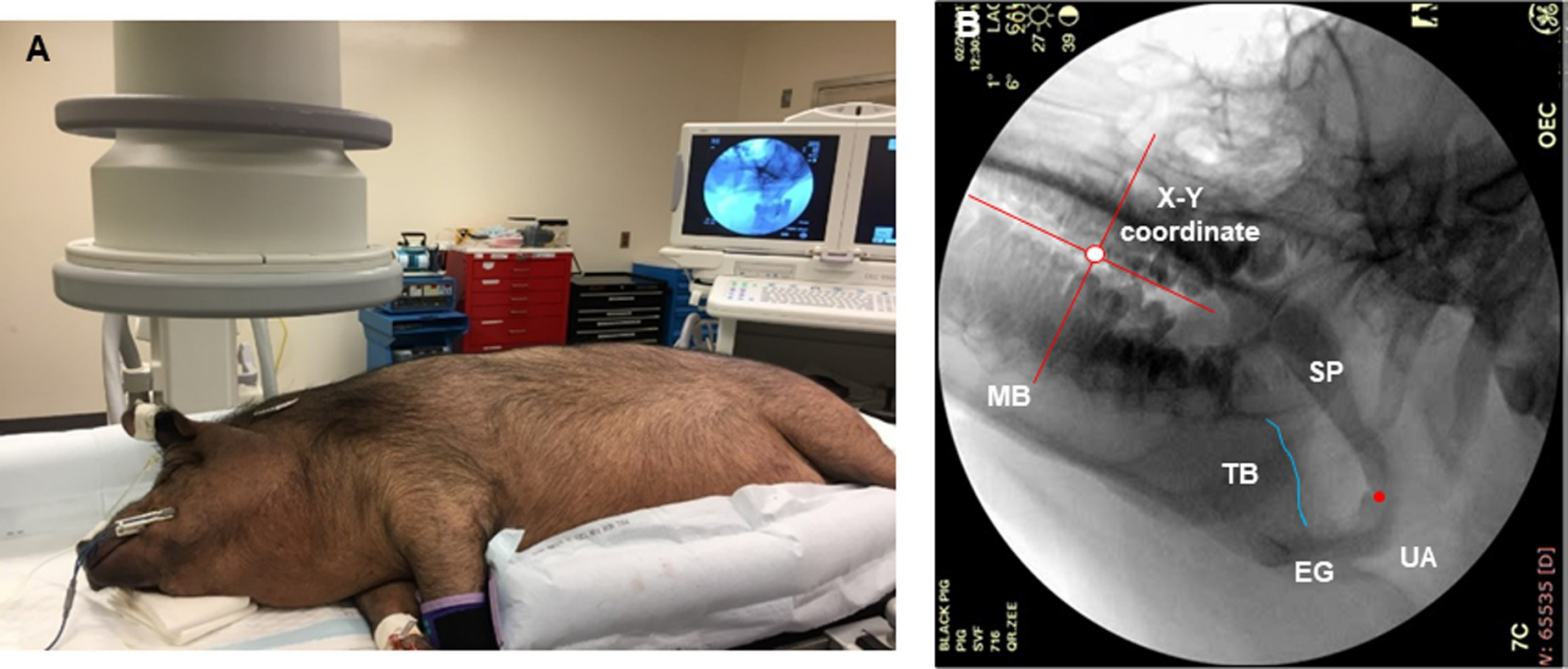
Sleep video-fluoroscopy (SVF). **A:** SVF imaging; **B:** Sagittal SVF image of oropharyngeal region. **MB:** mandible; **TB:** tongue base; **SP:** soft palate; **EG:** epiglottis; **AW:** airway. The blue line indicates the dorsal surface of the tongue base, and the red dot indicates the tip of the soft palate. The red cross indicates the X-Y coordinate axes, and the white dot indicates the origin of the coordinates.

3) SVF was performed while the pig was placed on the C-Arm table with 4 different positions (prone, back, lateral left and lateral right) at 30 frames/s using a GE C-Arm video-fluoroscopy unit (General Electric Co. MA, **Fig 3A**, Sleep video-fluoroscopy (SVF). **A:** SVF imaging; **B:** Sagittal SVF image of oropharyngeal region. **MB:** mandible; **TB:** tongue base; **SP:** soft palate; **EG:** epiglottis; **AW:** airway. The blue line indicates the dorsal surface of the tongue base, and the red dot indicates the tip of the soft palate. The red cross indicates the X-Y coordinate axes, and the white dot indicates the origin of the coordinates). A calibration metal pin was place on the pig’s head [12, 13].

### Image analyses

#### MRI–Fat composition

Fat and water images were separately reconstructed from Dixon MRI images. The fat fraction map was obtained as the ratio of the fat image to the sum of the water and fat images. Pixel values on the fat fraction map were thus calibrated to represent the fraction of signal in that pixel from fat (i. e. fat fraction ranges from 0% to 100%). Since the Dixon acquisition was obtained with isotropic voxels, image slices of the fat fraction map can be stacked as a volume and reformatted in multiple orientations to segment tissues such as the tongue and calculate the fat content in the segmented region. After calibration, those images were analyzed using Invivo6 (Anatomage, Inc., Santa Clara, CA). Invivo6 offers a 3-D view, and any location can be viewed from sagittal, coronal, and axial views. This function allowed us to determine the locations of the target structures of each specimen precisely.

The three regions (structures) of interest (ROI) are the tongue base, the soft palate, and the pharyngeal wall. The tongue base and the soft palate were analyzed using the coronal view (**Fig 1B**). To analyze each of the ROIs, the first step was to determine the boundaries of the ROI. From the sagittal view, the junction of the hard and the soft palates was set as the rostral border for both the tongue base and the soft palate; the caudal border of the soft palate was set as the point between the transition of the soft palate and the epiglottis; and the most caudal border of the tongue base was set at the end point of the tongue base. Navigating slices through the sagittal view allowed us to determine an accurate number of MRI slices of the coronal view containing the ROIs, then we sampled back each coronal slice (5mm each) to the points at the end of the tongue base and soft palate (**Fig 1B**). Therefore, a total of 8 slices with an interval of 5mm were selected for the tongue base and soft palate. For the pharyngeal wall, the mesial and distal borders were determined by establishing the first appearance and the last appearance of the pharyngeal wall in the sagittal view (**Fig 1C**). The mid-sagittal slice was first selected, and the other 2 slices were 5mm lateral and internal to the mid-sagittal slice respectively. Therefore, a total of 3 slices were measured for the pharyngeal wall. The measuring tool was a rectangular shape, which captured the maximum, minimum, mean, and area of adipose tissue in the enclosed areas automatically. The size of each rectangular box was maximized to capture the most area of the ROI while avoiding any air component in the measuring toolbox.

#### USE–Tissue stiffness

The USE data were collected in cine-loop format. This produces movie clips that span approximately 6 seconds for each elastographic measurement. At each location approximately 6 video clips were acquired. Custom-developed MATLAB software (The MathWorks, Natick, MA) extracted frames with independent elastography measurements from the video clips (generally one elastography frame per second). Each frame contained a colorized ROI in which the USE data were obtained by the instrument (**Fig 2B**); the color pixels of each ROI are related to the stiffness of the tissues. Custom-developed software automatically extracted a circular ROI from the center of the USE color box (**Fig 2C**). The diameter of the circular ROI was set equal to one-half of the average of the color box height and width. Additional custom MATLAB software mapped the image pixel colors to stiffness measurements according to a color scale produced by the ultrasound scanner (**Fig 2D**) [14]. For each video clip the USE values in the extracted ROIs were averaged over the 5–10 USE frames, producing a single average circular elastography ROI. The USE pixel values were then averaged over the video clips acquired in each region of the tongue and the left neck muscles.

#### SVF–Moving trajectories

Image J software (NIH, Bethesda, MD) was used to digitize SVF video clips, and only lateral projection images were measured. To define the movement direction and range of the tongue base and the soft palate during inspiratory and expiratory phases, the following steps were taken: 1) a static coordinate was set up. The Y-axis was the tangent line of the mesial surface of the 2^nd^ upper molar, and the X-axis was the line perpendicular to the Y-axis and ran through the occlusal plane of the upper molars (**Fig 3B**); 2) in each video clip, the dorsal surface of the tongue base and soft palate were outlined, and the caudal tip of the soft palate was taken as the point for the moving range (**Fig 3B**); 3) the positional changes of the two structures (dorsal surface of the tongue base and soft palate) were digitized and superimposed in every other video clip (sampling rate 15/s) for the two respiratory cycles in each animal; and 4) for separating the respiratory phases, the transition from inspiratory to expiratory phases was determined by the video clip showing the maximal airway expansion, and usually the duration of inspiratory was shorter than the expiratory phases in the ratio of 0.65:1.

### Reliability tests and statistics

Both intra- and inter- reader reliability tests were performed for MRI and SVF measurements. The same investigator (DL) measured the same 10 MRI slices and 15 SVF video frames twice at two separate times (10 days apart), and the second investigator (EW) performed the same image analyses on these MRI frames and SVF video clips. Wilcoxon signed rank tests showed no significant differences between the two measurements for both MRI and SVF images for inter-reader tests (p = 0.23, r^2^ = 0.99). The measurements of the intra-reader errors were calculated by Dahlberg’s equation [15] and the error was 0.72%.

Due to the exploratory nature of the study and the limited sample sizes in each of date sets, Kruskal-Wallis and Mann-Whitney non-parametric tests (SPSS, IBM^@^) were used to examine the differences of each measured parameters with the significant level of p < 0.05.

## Results

### MRI–Fat composition

**Fig 4:**
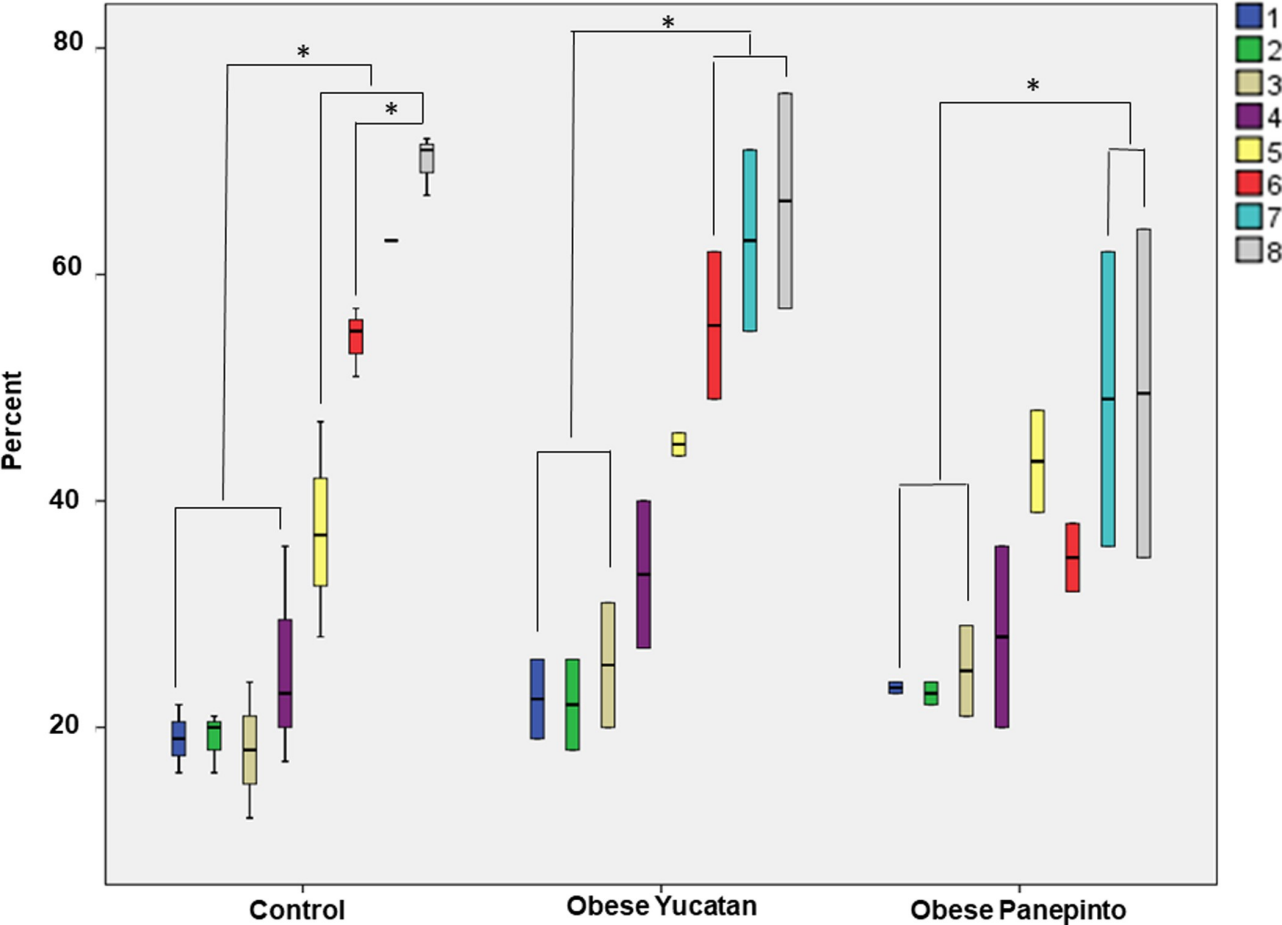
Boxplots showing the % of fat composition from the rostral to caudal tongue base in each group. The color boxes and numbers indicate the coronal regions of the tongue base from the rostral to caudal regions. Numbers 1–8 indicate eight coronal regions from the most rostral (#1) to the most caudal (#8) region of the tongue base with an interval of 5mm between regions. Asterisks indicate significant difference p < 0.05.

The % of fat composition in the tongue base was mapped against location to show a relationship between distribution of fat composition from anterior to posterior region of the tongue base in each group. The boxplot indicates that there was gradually increased amount of fat composition from the rostral to caudal regions in all three groups, and the tongue base 30-40mm caudal to the conjunction of the soft and hard palates had significantly higher fat composition than the region 5-15mm rostral to the conjunction (p < 0.05, **Fig 4,** Boxplots showing the % of fat composition from the rostral to caudal tongue base in each group. The color boxes and numbers indicate the coronal regions of the tongue base from the rostral to caudal regions. Numbers 1–8 indicate eight coronal regions from the most rostral (#1) to the most caudal (#8) region of the tongue base with an interval of 5mm between regions. Asterisks indicate significant difference p < 0.05). This trend was more significant in the controls and obese Yucatan than Panepinto minipigs. Both Yucatan groups started out at about 20% fat composition in rostral tongue base and sharply increased to about 60–70% in caudal tongue base. The trend of the obese Panepinto minipigs, however, showed a less degree of change in % of fat composition. It started out at about 25% in the rostral tongue base and gradually increased to about 50% in the caudal tongue base.

**Fig 5:**
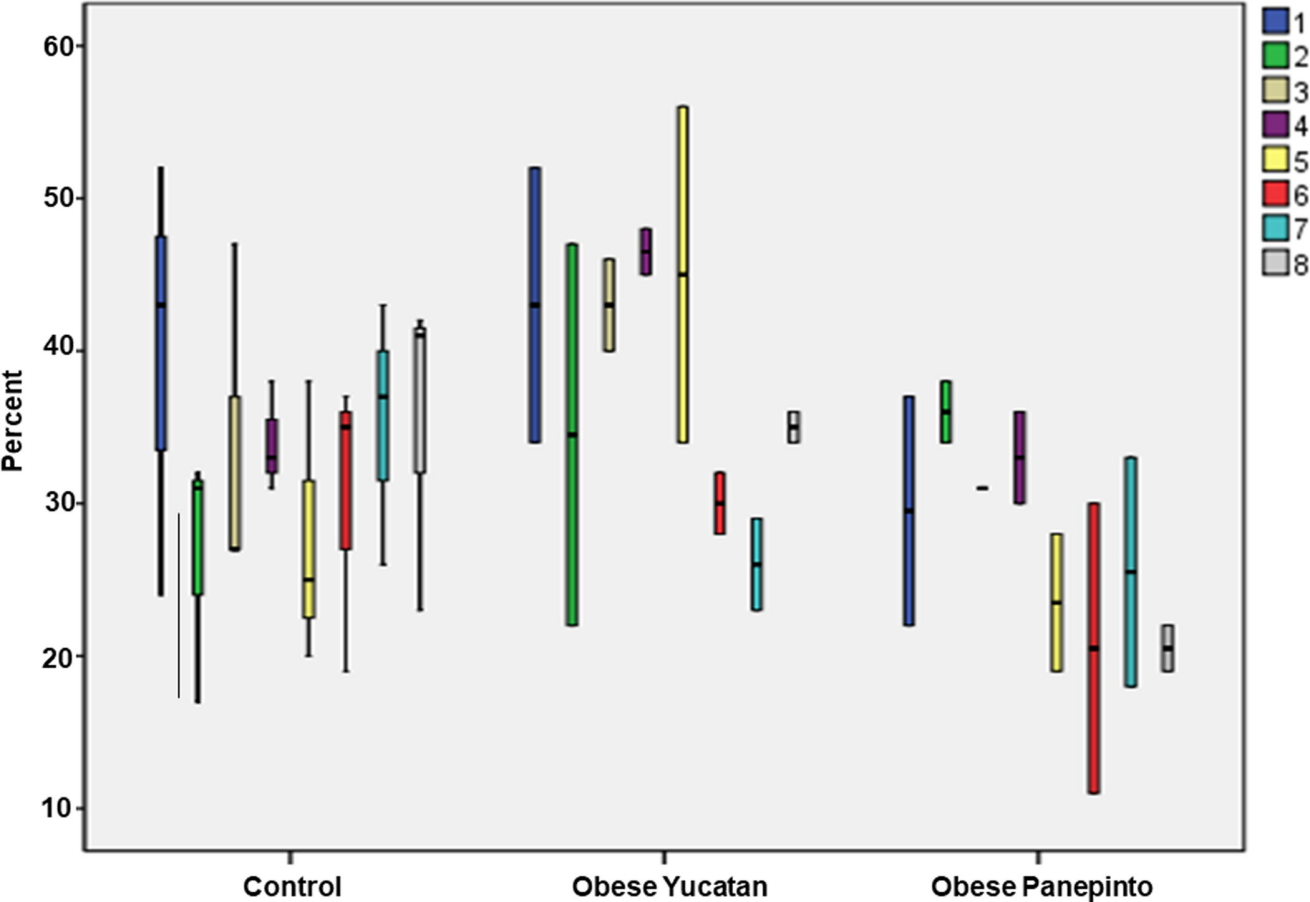
Boxplots showing the % of fat composition from the rostral to caudal soft palate in each group. The groups are the same as in **Fig 4**.

For the % of fat composition in the soft palate, while the control group showed less changes from the rostral to caudal regions, both obese groups showed a downward trend from the rostral to caudal regions. Overall, lower fat composition was seen in Panepinto as compared with the controls and obese Yucatan minipigs (**Fig 5**, Boxplots showing the % of fat composition from the rostral to caudal soft palate in each group. The groups are the same as in **Fig 4**).

**Fig 6:**
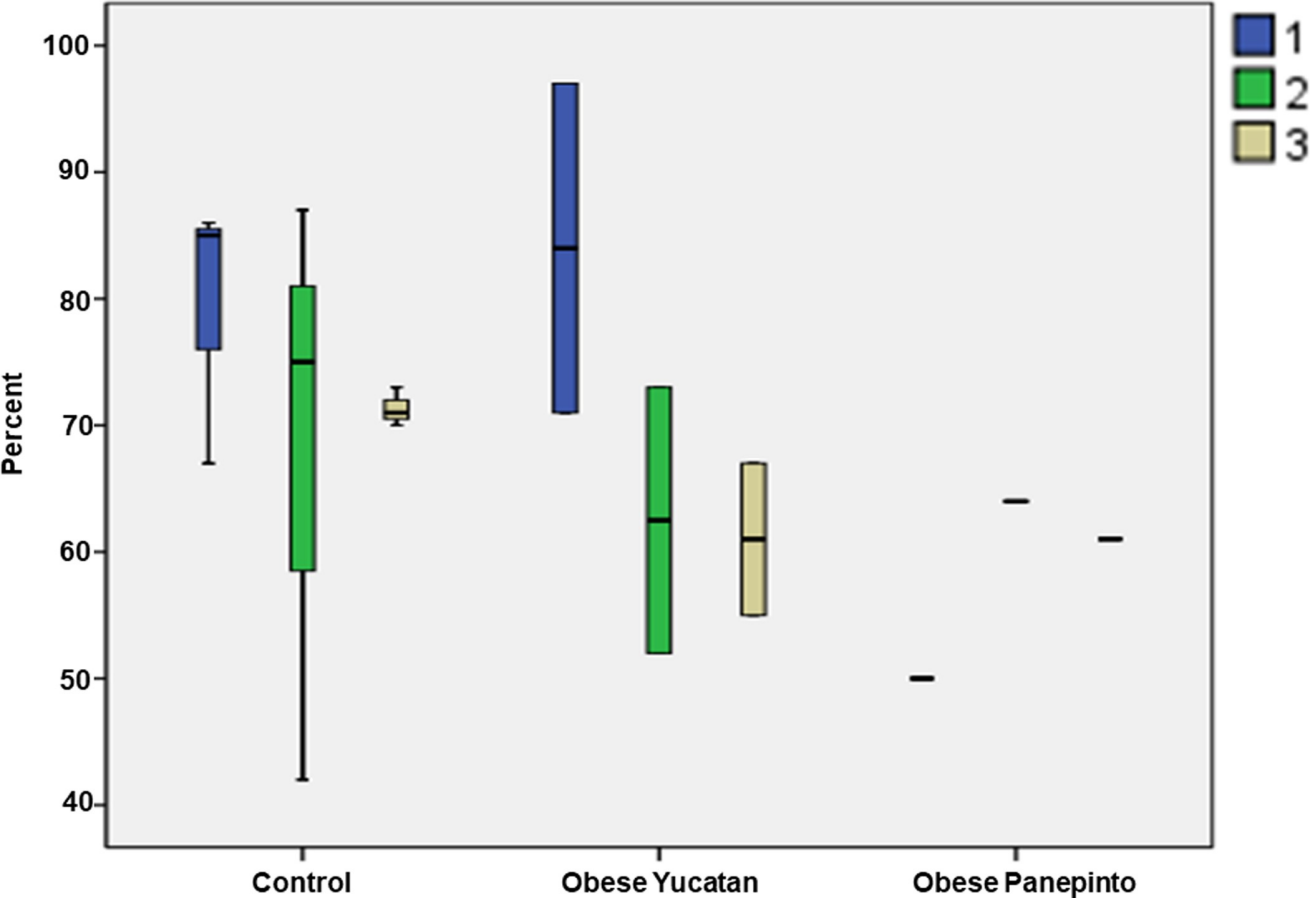
Boxplots showing the % of fat composition of the pharyngeal wall in each group. The color boxes and numbers indicate the sagittal regions of the pharyngeal wall from lateral, mid-sagittal and internal. Numbers 1–3 indicate 3 sagittal regions from lateral (#1), mid-sagittal (#2), and internal (#3).

The overall % of fat composition was significantly higher in the pharyngeal wall as compared with the overall values in the tongue base and soft palate, which was close to or even higher than the caudal tongue base and reached as high as 70–90%. Again, Panepinto minipig presented (data from one animal only) a lower value at the region 5mm lateral to the mid-sagittal region (**Fig 6**, Boxplots showing the % of fat composition of the pharyngeal wall in each group. The color boxes and numbers indicate the sagittal regions of the pharyngeal wall from lateral, mid-sagittal and internal. Numbers 1–3 indicate 3 sagittal regions from lateral (#1), mid-sagittal (#2), and internal (#3)).

### USE–Tissue stiffness

**Fig 7:**
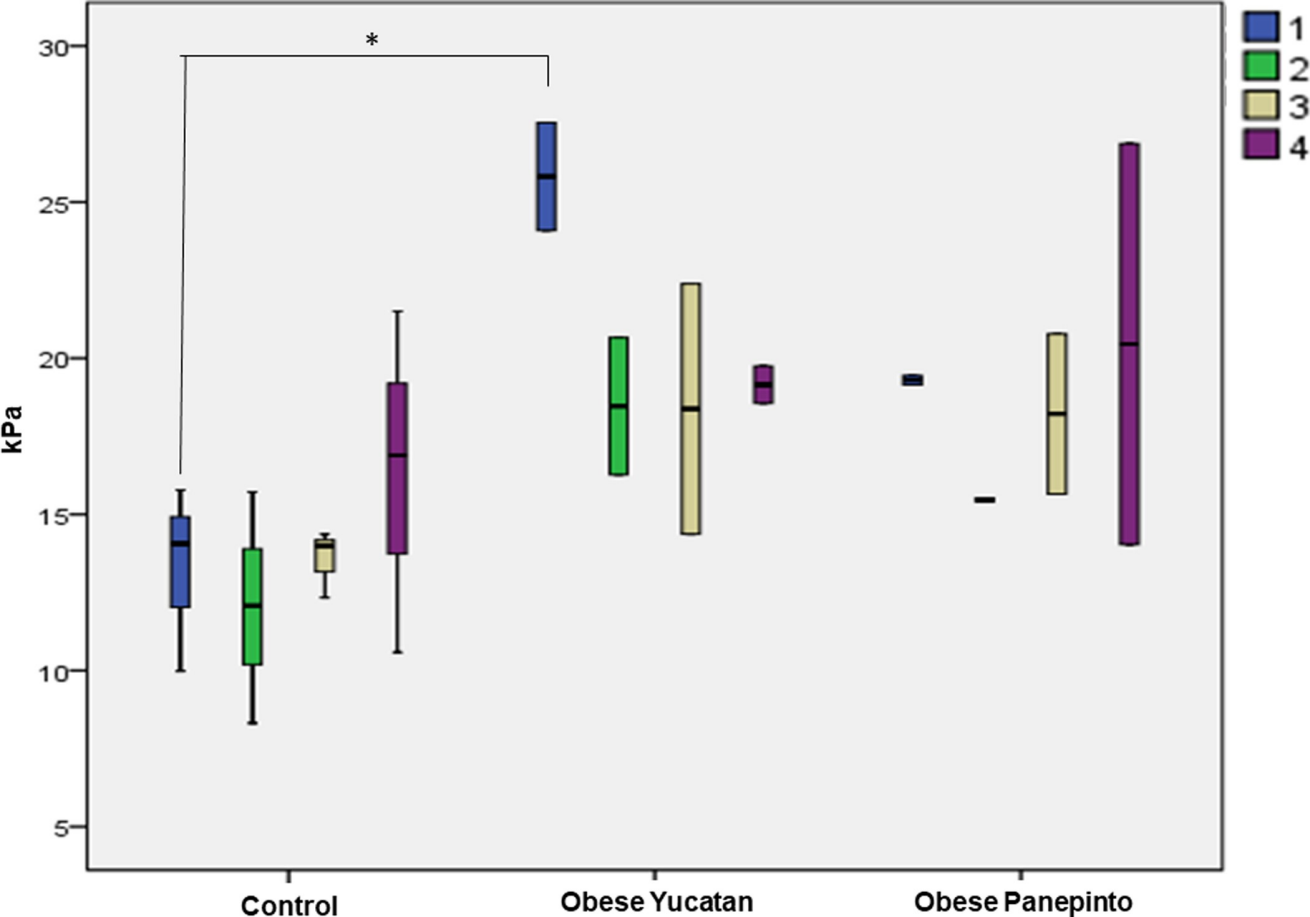
Boxplots showing the tissue stiffnesses of rostral, middle, and caudal tongue and the neck muscles by USE. The color boxes and numbers #1, #2, and #3 indicate rostral, middle, and caudal regions of the tongue, respectively, and #4 indicates neck muscles.

Overall, the obese minipigs presented higher stiffness values in all three regions of the tongue and neck muscles as compared to the controls, and the neck muscles presented the highest stiffness as compared to those of tongue regions in each group. There were no obvious differences between the 3 tongue regions in each group except the rostral tongue in Yucatan obese minipigs, which was significantly higher than that in Yucatan controls (**Fig 7**, Boxplots showing the tissue stiffnesses of rostral, middle, and caudal tongue and the neck muscles by USE. The color boxes and numbers #1, #2, and #3 indicate rostral, middle, and caudal regions of the tongue, respectively, and #4 indicates neck muscles).

### SVF–Respiratory movements

**Fig 8:**
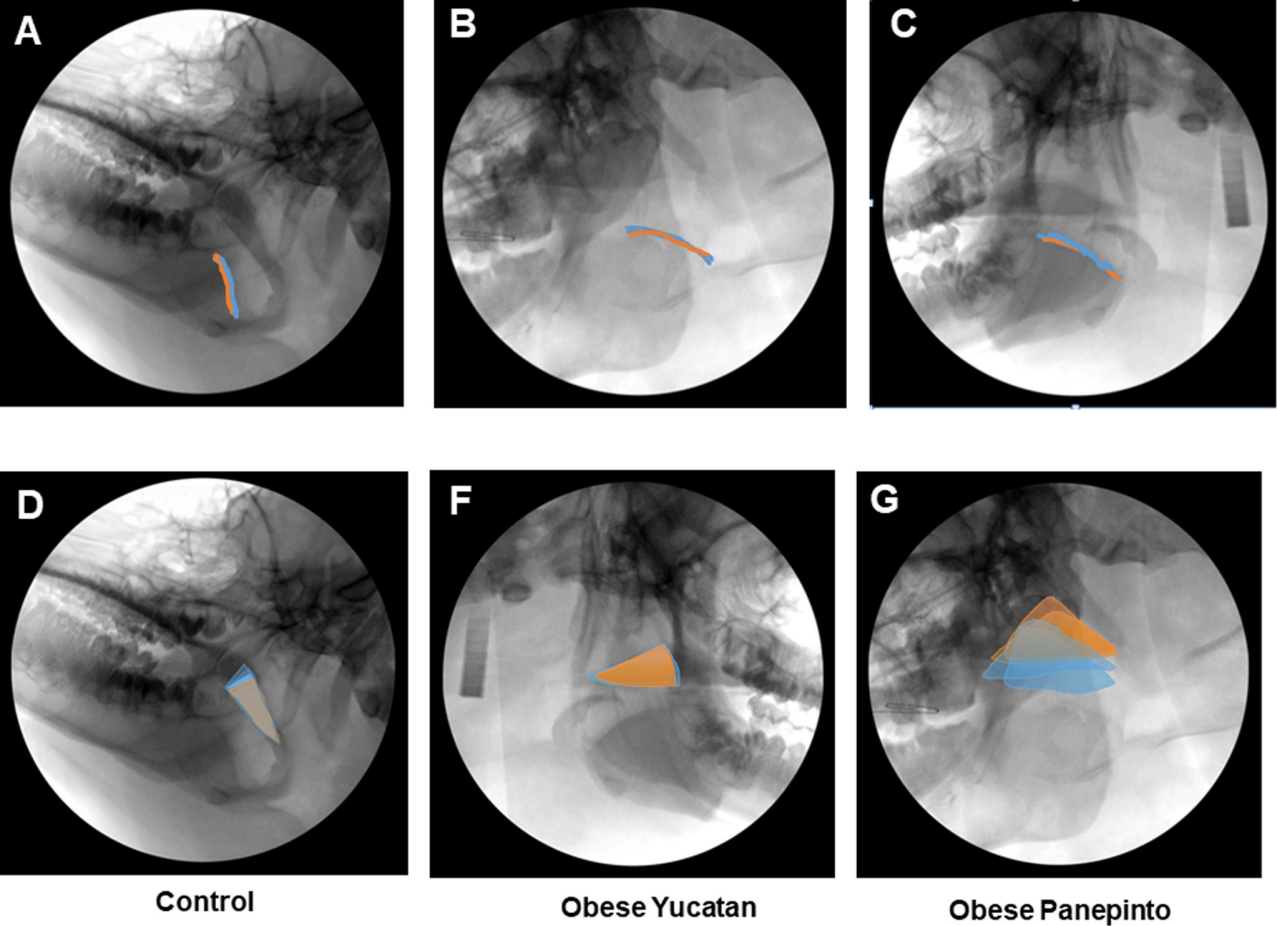
Superimposed moving trajectories of the dorsal surface of the tongue base (upper row) and tip of the soft palate (lower row) during inspiratory (blue) and expiratory (orange) phases during sedated sleep. All images were adjusted to have the lower border of the mandible parallel to the earth. **A, D:** non-obese Yucatan #716; **B, F:** Obese Yucatan #954; **C, G:** Obese Panepinto #10.

As shown in **Fig 8** (Superimposed moving trajectories of the dorsal surface of the tongue base (upper row) and tip of the soft palate (lower row) during inspiratory (blue) and expiratory (orange) phases during sedated sleep. All images were adjusted to have the lower border of the mandible parallel to the earth. **A, D:** non-obese Yucatan #716; **B, F:** Obese Yucatan #954; **C, G:** Obese Panepinto #10), the superimposed moving ranges of the dorsal surface of the tongue base were significantly smaller than these of the soft palate during respiration, and the component of its dorsal-ventral excursion (Y-axis) was much smaller than its rostral-caudal (X-axis) excursion. There was no difference between obese OSA and control minipigs, and between inspiratory and expiratory phases. However, the range of soft palate excursions in both rostral-caudal and dorsal-ventral directions during expiration were larger than those during inspiration in all three groups, and obese OSA Panepinto minipigs showed the largest moving range.

The respiratory moving range of the soft palate tip in both X and Y directions were small (2.33–3.67mm) in the controls, but significantly larger (4.02–7.50mm) in obese OSA Yucatan minipigs. In obese OSA Panepinto minipigs, the moving range in X direction was similar to those in obese OSA Yucatan minipigs, but the range in Y direction was highly significantly larger (30.38–31.68mm) than those in the other two groups (**Table 3**), which might contribute to significantly heavy snoring during expiration observed in both Panepinto minipigs during SVF imaging.

**Table 3:** Moving range of the soft palate tip (mm).

Groups	Pig #	X-axis	Y-axis
**Non-obese**	981	3.67	2.33
**Yucatans**	716	2.64	3.24
	**mean**	**3.16**	**2.79**
**Obese OSA**	930	4.02	6.39
**Yucatans**	954	7.20	7.50
	**mean**	5.61*	6.945*
**Obese OSA**	7	5.17	20.38
**Panepintos**	10	8.92	31.68
	**mean**	7.045*	26.03**

## Discussion

OSA affects up to 10% of children, 24% of adult men, and 9% of adult women, and it has been linked to fatigue-related accidents and cardiovascular diseases [16]. Obesity has been suggested to be one of the biggest risk factors for OSA. However, the mechanism behind it is still unknown. The size and tissue composition of the oropharyngeal structures can greatly affect OSA since they can impede the airflow and cause a partial or complete obstruction of the oropharyngeal airway. The goal of the present study is to shed light on the mechanism behind the blockage of the airway with a hypothesis that that an increased fat composition in oropharyngeal structures alters their tissue property and distorts their respiratory kinematics, thus leading to oropharyngeal airway collapse and predisposition to OSA.

### Large animal model for OSA study

While a number of animal models, such as rat, guinea pigs, rabbits, cats, and dogs, have been used for OSA study, minipigs have been chosen not only due to the similarities in the oropharyngeal airway regarding the architectures and tissue types, but also in tongue/soft palate shapes and sizes between minipigs and humans [17–20]. Most importantly, spontaneous OSA was identified in individual obese minipig [21] and was further validated in the two different breed of obese minipigs by our group [9, 22]. In addition, most physiological instrumentation for monitoring OSA, surgical, electrical, and ultrasonic interventions for treating OSA, and pharmaceutical testing are only suitable for large animals with spontaneous OSA. Therefore, these obese minipigs have a potential translational value as an ideal OSA animal model for a variety of OSA mechanism studies and the evaluation of emerging treatment strategies for OSA. Despite these advantages, three major differences of upper airway between humans and pigs in anatomy and physiology should be noted: 1) the respiration rate of the pig (25-30/min) [9] is about 100% faster than this in human (12-18/min) [23]; 2) unlike humans, the pig has no descending upper airway, but the orientation of the upper airway in the sleep position is similar to that of the human (**Fig 1C**); and 3) the tips of soft palate and epiglottis are close to each other or even overlapped (**Fig 3B**), while these two structures are separated widely in the vertical direction in humans.

### Fat deposition

Using 3 different imaging techniques (MRI, USE and SVF), the present study analyzed the images from these 3 sources in both obese OSA and non-obese/non-OSA control minipigs. The results shed some light into how the tissue properties and kinematics of the tongue base and soft palate relate to OSA and may lead to better understanding of pathophysiology for OSA. However, since no airflow data was simultaneously collected with these images, the associations between these imaging results and degrees of airway collapse and/or severity of OSA cannot be addressed by the present study.

The results from the MRI images suggest that percentage of fat composition increases from the middle region of the tongue base toward the most caudal region in all three groups. This result is consistent with an autopsy study in human cadavers [16]. The results also showed less fat deposition in the soft palate than in the tongue base and pharyngeal wall. However, the results were unable to demonstrate that obese OSA minipigs contained significantly more fat tissue in the tongue base, soft palate, and oropharyngeal wall than the controls as revealed in other studies in obese rats and humans [24, 25]. In contrast, obese OSA Panepinto minipigs even presented less fat tissue as compared with the controls and obese OSA Yucatan minipigs, particularly in the caudal region of the tongue base and soft palate (**Figs 5 and 6**). Three reasons may be considered for these discrepancies. First, there is a great age difference between Yucatan and Penapinto minipigs (8–11 months *vs* 78 months). Although the fat tissue in the tongue increases with age in humans [26], this may not be the case in minipigs. In addition, the fat composition greatly varies in different breeds of pigs [27]. Second, since the fat depositions were calculated as a percentage, rather than the actual amount, the size of the tongue could have confounded the measurements as obesity usually leads to a large-sized tongue [28]. Third, no same-aged non-obese control of Panepinto minipigs were included in the present study due to the unavailability. This limitation might lead to uncertainty about why the aged obese Panepinto minipigs presented lower fat compositions in these oropharyngeal structures.

### Tissue stiffness

The USE results revealed that overall, the tissue stiffness of the tongue was higher in the obese OSA than control minipigs, significantly in the rostral region in obese OSA Yucatan minipigs (**Fig 7**). These results are contradicted with the findings of more fat composition in obese rats and humans [24, 25], as more fat infiltration in the tongue should lead to less tissue stiffness in the tongue [29]. However, the present findings are in agreement with a USE study in awake OSA patients, which found that the mid-sagittal tongue of OSA patients had significantly higher tissue stiffness than those in controls during normal breathing, and the highest value was in the tongue base during Mϋller’s maneuver [30]. The tongue comprises the major dilator muscle of the oropharyngeal airway, i.e., genioglossus, and the tongue dilator muscle is synergically active with multiple oropharyngeal muscles such as palatal, hyoid, and pharyngeal muscles. While the components of the muscle and connective tissue contribute to the tissue stiffness, the higher muscle tone or isometric contraction caused by neural drive may also contribute to the tissue stiffness. Some electromyographic studies have also demonstrated that the motor units presented longer duration and larger size index in OSA patients [31]. Although MRI results show a trend that the fat composition gradually increases from rostral to caudal tongue base, no corresponding decreased trend of tissue stiffness was found in the USE analyses. In contrast, the rostral tongue of obese Yucatan minipigs presented the highest stiffness value. Therefore, the tissue components may not be the major determinant factor for the tissue stiffness measures in these minipigs. Since above-cited human OSA studies presented either tongue fat deposition or tissue stiffness alone, direct correlation between these features is unknown. Therefore, combining both approaches in human subjects is the future direction to better understand the relationship of the tissue composition and stiffness in the tongue.

### Respiratory movements

As expected, all obese OSA minipigs have larger respiratory excursions of the soft palate, either in the entire soft palate or its tip, and the excursion ranges are larger in expiratory than inspiratory phases. With an audible heavy snoring, these excursion ranges become significantly larger as seen in obese Panepinto minipigs, especially in the dorsal-ventral direction. However, the respiratory excursion ranges of the tongue base are small and show no differences among the three groups and between respiratory phases. It has been documented that the soft palate rises to touch the posterior pharyngeal wall, thus closing the nasopharynx to regulate airflow through nose and/or mouth [32]. The present study further demonstrates that the kinematics of the soft palate plays an important role in respiration, and its excursion range is larger in expiration than inspiration, most significantly in dorsal-ventral excursion. This study also suggests that the soft palate may be much more deeply involved in the genesis of snoring and OSA than the tongue base. This may explain why the uvulopalatopharyngoplasty (UPPP) which shortens and stiffens the soft palate by partially removing the uvula and reducing the edge of the soft palate, has been a popular surgical intervention to treat OSA in the past. However, due to the suspendable, movable, and flexible nature of the soft palate, the focus of surgical intervention for OSA treatment has shifted to the tongue base in recent years [33, 34].

### Limitations of the study

The limitations in the present study include: 1) the small sample sizes in each type of minipig, particularly the lack of the same aged controls of Panepinto minipigs due to the source unavailability. Therefore, the observed differences in Panepinto minipigs could be from the different breeds of minipigs. However, given the fact that Panepinto minipigs are a Yucatan crossbreed [35], and obese Panepinto had similar BMI to obese Yucatan minipigs. It could be reasonably speculated that the observed differences between normal Yucatan and Panepinto minipigs were most likely resulted from obesity and/or OSA; 2) as described in the methods, all recordings of the present study were performed under sedated sleep, instead of natural respiration in consciousness or sleep. Fortunately, the confounding effect of sedation and anesthesia on respiration has been proven to be minor [36–38], and the physical parameters between sedated and natural sleep present clear similarity in these normal and obese minipigs [9]. Therefore, this limitation could be considered minor but still needs to be confirmed during natural respiration when the technique becomes available; 3) no simultaneous airflow data was collected, thus the outcomes cannot be related to the degree of airway collapse; 4) the positional relationship of the tips of the soft palate and epiglottis differs between the pig and human; and 5) the procedures applied in the present study were invasive in nature and could not be directly applied in human subjects. However, given the original purpose of the study was to develop and validate a large animal model with spontaneous OSA, the potential relevance to OSA occurrence needs to be explored and characterized.

## Conclusions

The takeaway points of the present study are: 1) the fat composition is highest in the posterior 1/3 of the tongue base, regardless of the presence of obesity and OSA, and pharyngeal wall presented the highest fat composition as compared with the tongue base and soft palate; 2) obese OSA minipigs have stiffer tongue tissue than the controls, particularly in the rostral region of the tongue, which may result from altered neuromuscular drive; 3) the moving range of the soft palate is significantly extensive in obese OSA than control minipigs. This increased range may have a compensatory effect on the potential oropharyngeal airway restriction or collapse.
